# Cardiac Manifestation in Wilson Disease: Results of a 9‐Year Prospective Cohort

**DOI:** 10.1002/jmd2.70106

**Published:** 2026-06-25

**Authors:** S. Quick, M. Cybularz‐Bednarek, L. Wiegand, K. Zhang, K. Ibrahim, M. Christoph, J. Hamann, A. Linke, F. M. Heidrich

**Affiliations:** ^1^ Department of Internal Medicine I, Cardiology, Angiology and Intensive Care, Klinikum Chemnitz gGmbH Medical Campus Chemnitz of the TUD Dresden University of Technology Dresden Germany; ^2^ Department of Internal Medicine and Cardiology, Herzzentrum Dresden TUD Dresden University of Technology Dresden Germany; ^3^ Department of Cardiology, Angiology and Intensive Care Medicine Deutsches Herzzentrum der Charité Berlin Germany

**Keywords:** cardiac involvement, follow‐up questionnaire, longitudinal study, patient‐reported outcomes, symptom assessment, Wilson disease

## Abstract

Wilson disease (WD) is a rare autosomal recessive disorder. Although hepatic and neurologic manifestations are characterized, long‐term cardiac outcomes remain poorly defined. We report the first longitudinal study systematically assessing cardiac symptoms and events over nearly a decade in WD. In 2016, 61 WD patients underwent prospective cardiac evaluation including echocardiography, MRI and 24‐h ECG. Nine years later, we re‐contacted all surviving participants by telephone and emailed standardized questionnaires. Baseline characteristics of respondents and non‐respondents were compared to assess selection bias; mortality data were obtained from family interviews and medical records. Outcomes were available for 31 of 61 patients (27 interviewed, 4 deceased; no cardiac‐related deaths) after a mean follow‐up of 9.1 years. Respondents did not differ from those lost to follow‐up, except for fewer disease exacerbations. General and cardiac health remained unchanged in about half (48% and 52%), worsened in ~40%, and improved in ≤ 15%. Palpitations were frequent (70%), whereas dizziness (26%), syncope (7%), and leg oedema (15%) were infrequent and comparable to general‐population estimates. Daily activity was unrestricted in 44%, mildly to moderately restricted in 52% and severely restricted in 4%. Only six patients (22%) remained in routine cardiology care. Baseline imaging, strain, autonomic and laboratory parameters did not predict symptoms at follow‐up. Over 9 years, cardiac symptoms in WD were common but mostly mild to moderate. These findings support a symptom‐guided cardiological assessment within interdisciplinary care. Whether systematic screening of asymptomatic patients is warranted remains uncertain and requires dedicated prospective study.

## Synopsis

This 9‐year follow‐up study shows that cardiac symptoms in Wilson disease are common but mostly mild to moderate; serious cardiovascular events are rare and baseline cardiac or autonomic abnormalities do not reliably predict later symptoms, supporting symptom‐guided cardiological assessment within interdisciplinary care.

## 1 Introduction

Wilson disease (WD) is a rare autosomal recessive disorder characterised by defective copper metabolism, leading to copper accumulation predominantly in the liver, brain and cornea [1]. While hepatic and neurological involvement are well documented, cardiac and autonomic nervous system manifestations have received comparatively limited attention [2, 3]. Initial studies, including our investigations in 2018 and 2019, highlighted subtle cardiac involvement—reduced ventricular function, diastolic dysfunction, increased myocardial fibrosis on cardiac MRI and autonomic dysfunction indicated by reduced heart rate variability (HRV) [4, 5].

Given these findings, understanding the longitudinal progression of cardiac involvement and autonomic dysfunction in WD patients is essential. Hence, this follow‐up study aimed to comprehensively evaluate the progression of cardiac and autonomic manifestations over a decade in our original WD patient cohort.

## 2 Methods

### 2.1 Study Design and Original Cohort

This investigation is a prospective longitudinal follow‐up of a previously characterised single‐centre cohort of patients with WD. The original study, conducted in 2016, comprised 61 consecutive patients with an established diagnosis of WD who underwent comprehensive cardiac phenotyping, including cardiac magnetic resonance (CMR) imaging, echocardiography with myocardial strain analysis, 24‐h Holter electrocardiography with HRV analysis, and laboratory testing. Baseline disease severity was quantified using the Unified Wilson Disease Rating Scale (UWDRS), and the clinical phenotype was classified according to the Ferenci criteria (hepatic, neurologic or psychiatric). The baseline methodology and results have been reported in detail elsewhere [4, 5].

### 2.2 Follow‐Up Assessment

In 2025—a mean of 9.1 years after baseline—all surviving members of the original cohort were re‐contacted by telephone and by emailed questionnaire. A standardised survey captured (i) self‐rated change in overall health and in cardiac health (shortness of breath/exercise intolerance) on a five‐point scale, (ii) pre‐specified cardiac symptoms (palpitations, dizziness, syncope and leg oedema), (iii) the functional impact of cardiac symptoms on daily activities, (iv) hospitalisation history, (v) adherence to WD‐specific therapy and (vi) frequency of cardiology follow‐up. For patients who had died, vital status and cause of death were ascertained from family interviews and review of available medical records.

### 2.3 Definition of Analysis Groups

Three groups were defined. The *original cohort* comprised all 61 patients assessed in 2016. The *follow‐up subgroup* comprised the 27 patients successfully re‐interviewed in 2025. The *lost‐to‐follow‐up group* comprised the remaining 34 patients (deceased, declined, unreachable or with outdated contact information). To assess potential selection bias, baseline characteristics of the follow‐up subgroup were compared with those of the lost‐to‐follow‐up group.

### 2.4 Statistical Analysis

Continuous variables are presented as mean (standard deviation [SD]) and categorical variables as counts and percentages; available‐case denominators are reflected in the tables. Normality of continuous variables was assessed with the Shapiro–Wilk test. Normally distributed variables (e.g., age, disease duration and echocardiographic strain) were compared using the unpaired Student's *t*‐test, and non‐normally distributed variables (e.g., UWDRS scores, NT‐proBNP, troponin T) using the Mann–Whitney *U* test. Categorical variables (sex, disease exacerbation, midwall late gadolinium enhancement) were compared using Fisher's exact test.

To examine whether minor baseline cardiac, autonomic or biochemical abnormalities predicted patient‐reported symptoms at follow‐up, Spearman rank correlation coefficients (*ρ*) were calculated between each pre‐specified baseline parameter and each patient‐reported outcome within the follow‐up subgroup; outcomes were scaled so that higher values indicated worse health or more severe symptoms. A correlation was considered potentially meaningful when |*ρ*| exceeded 0.3. Given the exploratory nature and the number of comparisons, no formal correction for multiple testing was applied; the total number of tests is reported and nominally significant findings are interpreted with caution. The association between baseline UWDRS and current health status was assessed using Spearman correlation, and that between health deterioration and Ferenci phenotype using the *χ* ^2^ test. A two‐sided *p* < 0.05 was considered significant. Analyses were performed using Python 3.11 (SciPy, pandas).

### 2.5 Ethics

This study was approved by the local ethics committee of the Technische Universität Dresden (BO‐EK‐212052024). All participants provided informed consent prior to inclusion.

### 2.6 Artificial Intelligence Generated Content (AIGC)

As non‐native English speakers, the authors used AIGC tools (such as ChatGPT [OpenAI]) solely for assistance with English phrasing and language editing, and to support optimisation of figures for presentation; no AI tool was used for data analysis or scientific interpretation, and all scientific content and final approval remained the responsibility of the authors.

## 3 Results

### 3.1 Follow‐Up Rate and Baseline Comparability

From the original cohort of 61 participants, 44% (27/61) were successfully interviewed after a mean time of 9.1 years. A total of 7% (4/61) were confirmed deceased (no cardiac‐related deaths), 15% (9/61) could not be reached, 7% (4/61) declined and 28% (17/61) lacked current contact information.

Baseline clinical characteristics of the follow‐up subgroup, the original cohort and the patients lost to follow‐up are presented in Table 1. The follow‐up subgroup did not differ significantly from the patients lost to follow‐up with respect to sex, age, disease duration or UWDRS scores (all *p* ≥ 0.29). The only significant baseline difference was a lower frequency of documented disease exacerbations in the follow‐up subgroup (12.5% vs. 43.8%, *p* = 0.018), suggesting that patients with a more active disease course were somewhat underrepresented among respondents. Baseline CMR, echocardiographic strain, laboratory and HRV parameters were comparable between the two groups (all *p* ≥ 0.03; Table S1), with only TAPSE marginally lower in the follow‐up subgroup (22.9 vs. 24.8 mm, *p* = 0.033).

**TABLE 1:** Baseline clinical characteristics of the original cohort, the follow‐up subgroup and the patients lost to follow‐up.

Variable	Original cohort (*n* = 61)	Follow‐up subgroup (*n* = 27)	Lost to follow‐up (*n* = 34)	*p*
Sex				
Male	31 (50.8%)	13 (48.1%)	18 (52.9%)	0.799
Female	30 (49.2%)	14 (51.9%)	16 (47.1%)	
Age (years)	44.3 (15.2)	44.6 (11.7)	44.0 (17.7)	0.885
Disease characteristics				
Disease duration at study entry (years)	24.9 (14.7)	24.8 (14.9)	21.3 (14.7)	0.385
Exacerbation during disease course	18 (29.5%)	3/24 (12.5%)	14/32 (43.8%)	0.018
UWDRS score				
Total	11.0 (19.2)	7.4 (11.8)	13.8 (23.2)	0.654
Neurologic	5.3 (13.9)	1.7 (3.1)	8.0 (17.9)	0.433
Internal	2.0 (1.9)	1.7 (1.6)	2.3 (2.1)	0.295

*Note:* Data are presented as mean (SD) or *n* (%) unless otherwise stated. *p*‐values compare the follow‐up subgroup with the lost‐to‐follow‐up group (Fisher's exact test for categorical variables; Student's *t*‐test or Mann–Whitney *U* test for continuous variables).

Abbreviation: UWDRS, Unified Wilson Disease Rating Scale.

Hospitalisations were relatively common: 26% (7/27) reported multiple admissions and 22% (6/27) a single admission. Only 4% (1/27) had a cardiovascular‐related hospitalisation (atrial‐fibrillation ablation).

### 3.2 Long‐Term Health Changes and Symptom Profile

**FIGURE 1:**
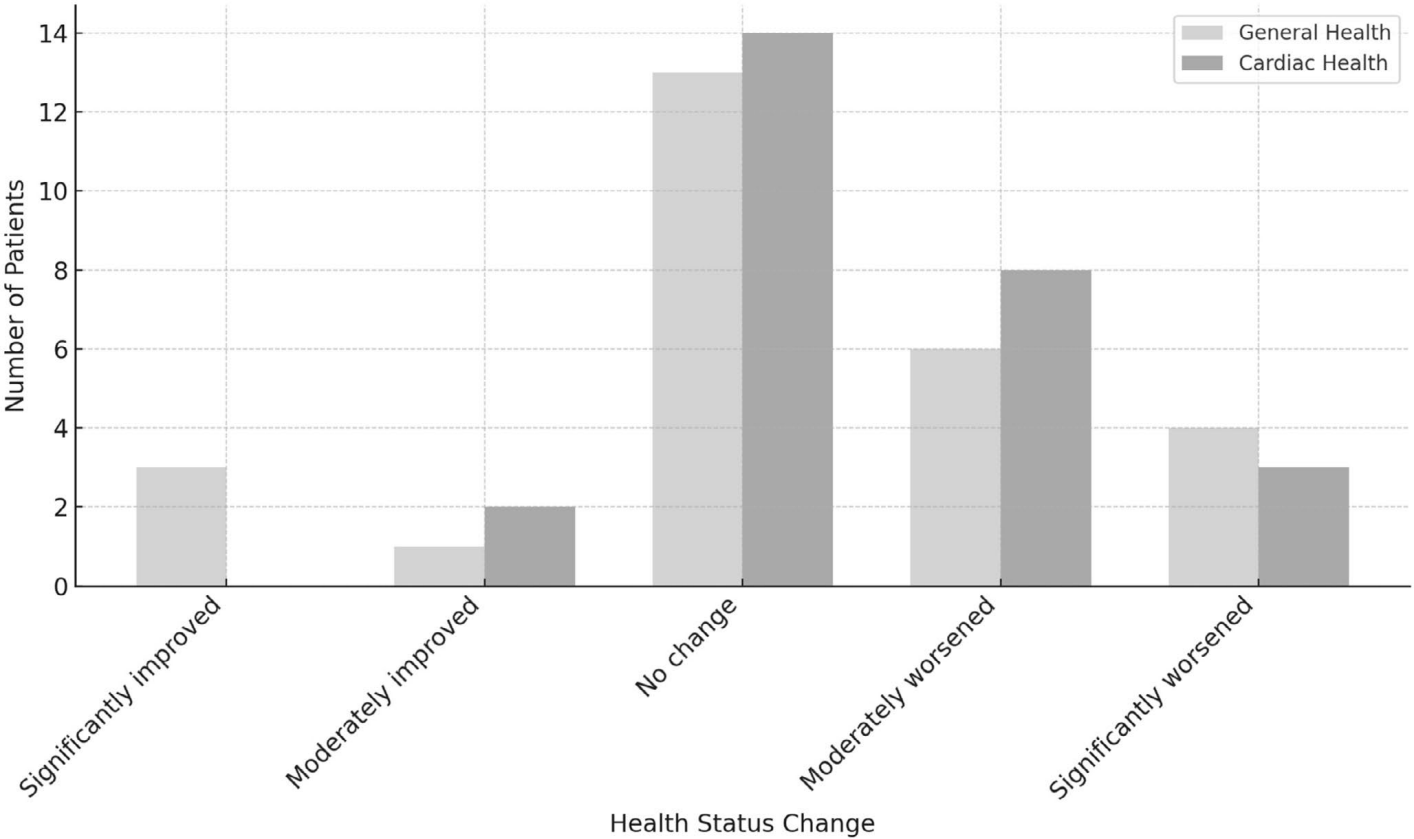
Comparison of self‐reported changes in general health and cardiac health over a 9‐year follow‐up period in patients with Wilson disease. Responses to both questions were categorised using the same 5‐point scale. Light grey bars represent general health; dark grey bars represent cardiac health.

Figure 1 summarises patient‐reported changes over 9 years in general health and a combined ‘cardiac health’ measure (shortness of breath/exercise intolerance) for the 27 respondents. Stable health was reported by 48% (13/27) for general health and 52% (14/27) for cardiac health. Worsening occurred in 37% (10/27) of general health and 41% (11/27) of cardiac health, whereas improvement was noted in only 15% (4/27) for general health and 7% (2/27) for cardiac health.

No significant association was observed between baseline UWDRS or the Ferenci phenotype and current health status. Spearman's *ρ* between health deterioration and UWDRS was 0.28 (*p* = 0.46); the *χ* ^2^ test of deterioration versus phenotype yielded *χ* ^2^(6) = 5.66 (*p* = 0.47).

**FIGURE 2:**
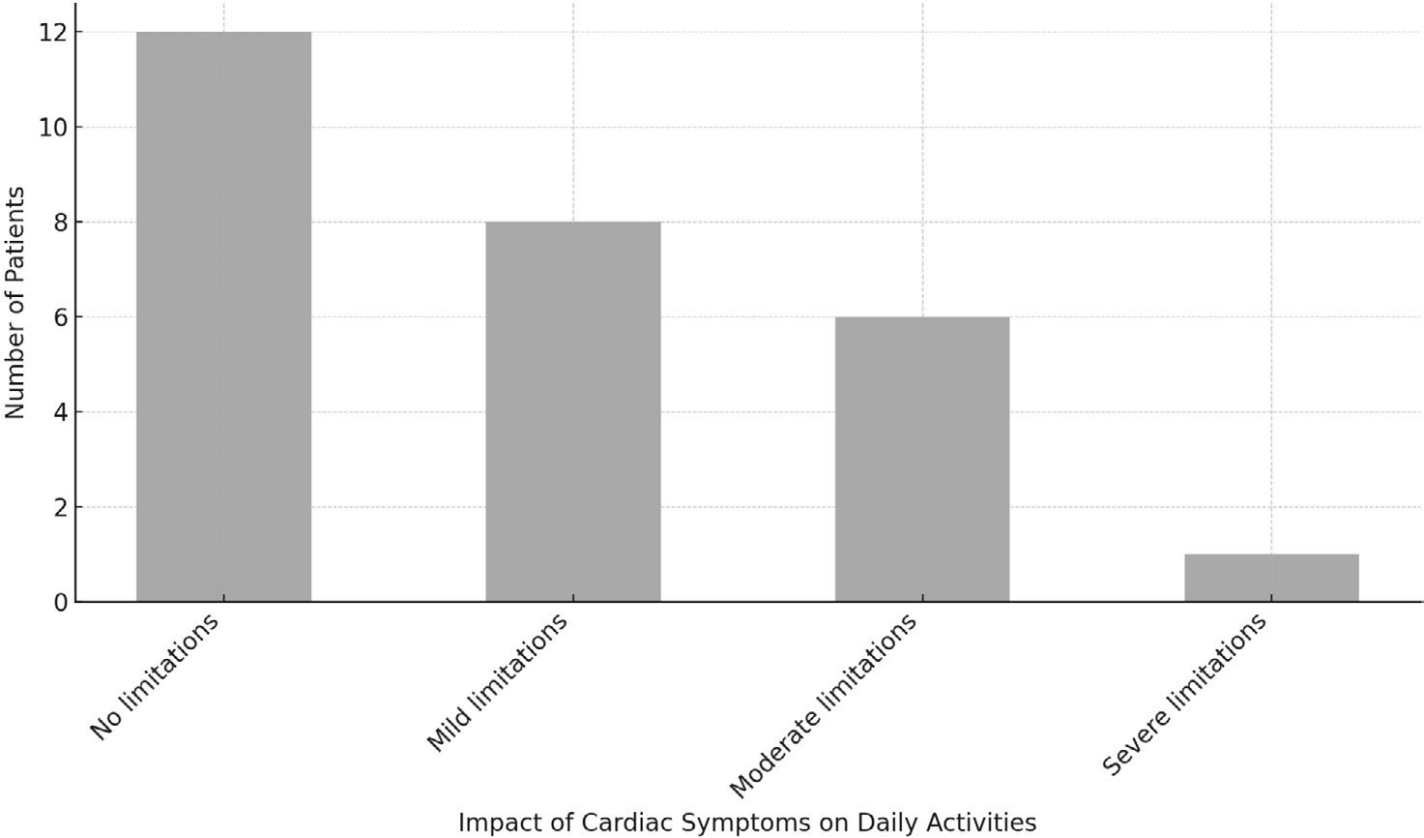
Frequency of specific cardiac‐related symptoms at 9‐year follow‐up.

Palpitations were reported by 70% (19/27)—63% (17/27) rarely or occasionally and 7% (2/27) frequently. Dizziness occurred in 26% (7/27), fainting in 7% (2/27) and leg oedema in 15% (4/27) (Figure 2).

**FIGURE 3:**
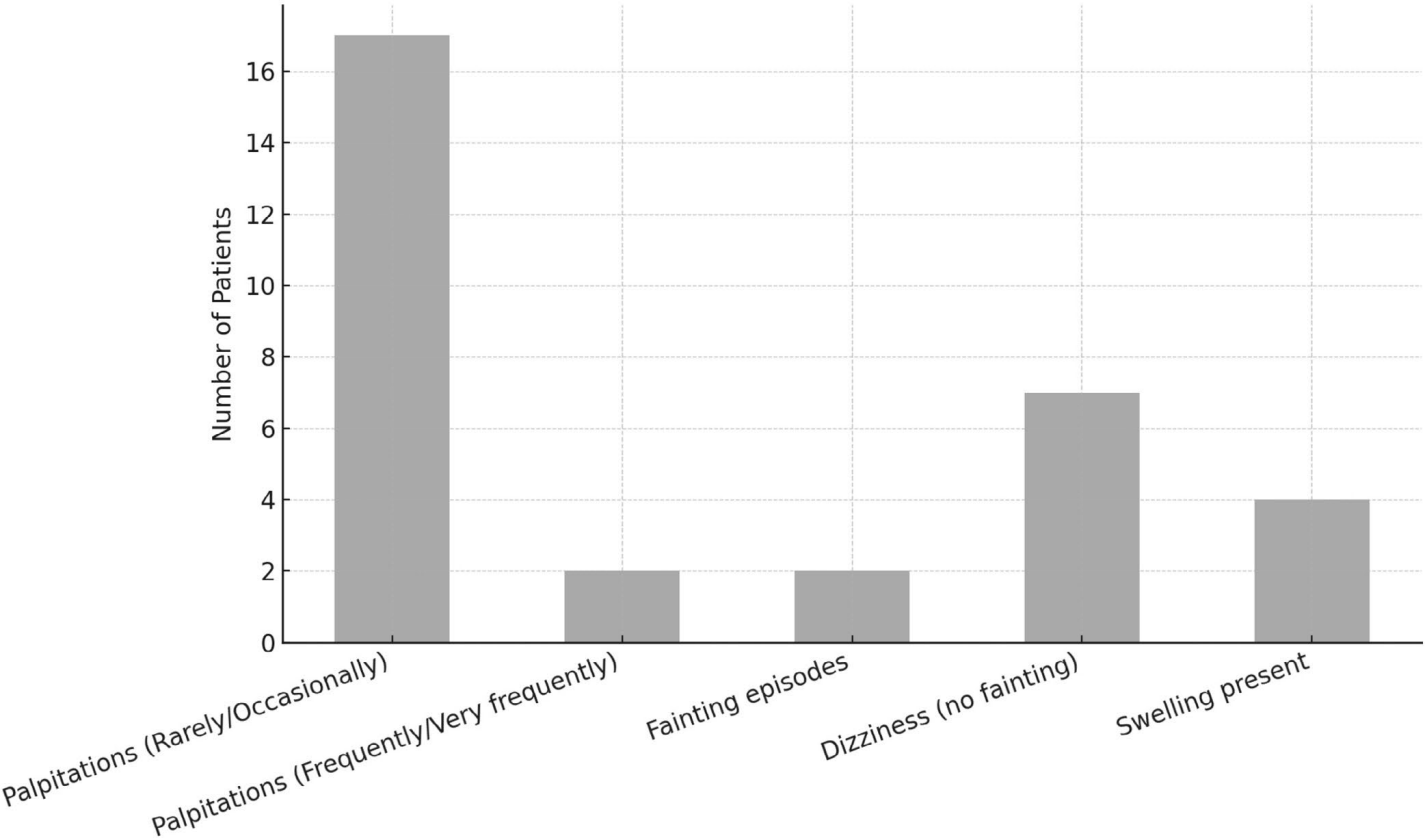
Impact of cardiac symptoms on daily activities in Wilson disease patients at 9‐year follow‐up.

Overall, most patients felt restricted by cardiac symptoms in daily activities. Figure 3 shows that 44% (12/27) reported no limitations, 30% (8/27) mild, 22% (6/27) moderate and 4% (1/27) severe limitations.

### 3.3 Association Between Baseline Parameters and 9‐Year Symptoms

To examine whether the minor cardiac and autonomic abnormalities identified at baseline predicted patient‐reported symptoms 9 years later, we correlated all pre‐specified baseline parameters with the follow‐up outcomes (Table S2). Of the 68 tested associations, only three reached nominal significance (*p* < 0.05): midwall late gadolinium enhancement with activity limitation (*ρ* = −0.54, *p* = 0.02) and with palpitation frequency (*ρ* = −0.46, *p* = 0.05) and right ventricular endocardial global longitudinal strain with general health change (*ρ* = −0.42, *p* = 0.04). All three were in a counter‐intuitive (negative) direction and are most parsimoniously explained by chance and the small number of patients with midwall LGE (*n* = 5); none remained significant after accounting for multiple testing.

Beyond modest links between dyspnoea and autonomic dysfunction (SDNN) and between activity limitation and selected strain metrics, we found no meaningful correlations (|*r*| > 0.3) between the pre‐specified cardiac symptoms and any objective measure recorded at baseline in 2016—including left and right ventricular ejection fraction, ventricular volumes, myocardial mass, echocardiographic strain, UWDRS scores or laboratory tests (NT‐proBNP, troponin T, total and non‐caeruloplasmin‐bound serum copper, ALT, AST). Current symptom burden did not align with the structural cardiac, biochemical or autonomic parameters measured 9 years earlier.

### 3.4 Cardiology Follow‐Up

Regular cardiology care was maintained by only 22% (6/27) of patients, with pathological findings reported in 50% (3/6) of these examinations.

## 4 Discussion

This 9‐year follow‐up—the first to prospectively track both cardiac symptoms and cardiovascular events in WD—offers insight into long‐term cardiac health in WD and confirms that serious cardiac outcomes (cardiac‐related death or hospitalisation) are rare.

Because outcomes could be ascertained for fewer than half of the original cohort, the representativeness of the follow‐up subgroup warrants careful consideration. Reassuringly, the 27 re‐interviewed patients did not differ from the 34 patients lost to follow‐up with respect to sex, age, disease duration, UWDRS scores or any baseline CMR, echocardiographic strain, laboratory or HRV parameter (Table 1; Table S1). The single exception was a lower frequency of documented disease exacerbations among respondents (12.5% vs. 43.8%). If anything, this indicates that patients with a more active disease course were somewhat underrepresented at follow‐up, which would bias our results towards an *underestimation* rather than an overestimation of long‐term cardiac symptom burden. The overall benign picture is therefore unlikely to be an artefact of preferential retention of healthier patients.

Hospitalisations were frequent, but only one was cardiac—a single atrial‐fibrillation ablation, a procedure too common in the general population to attribute specifically to WD despite its modestly increased atrial‐fibrillation risk (29% higher) [6, 7]. This pattern is consistent with the multi‐organ nature of WD, where hepatic and neurological complications often dominate clinical management [8, 9]. The near‐absence of cardiac‐related admissions or deaths during 9 years reinforces earlier reports of a low incidence of overt heart disease in WD [6]. Vigilance remains essential, however, because sudden cardiac death has been documented: the Paris reference centre recorded three unexplained deaths among 300 young WD patients, two probably caused by ventricular arrhythmias [10, 11, 12].

The general health status of participants showed a variable course, with more than half reporting moderate to significant deterioration, comparable to other studies [13]. Although previous studies reported worse general health in neurologic than hepatic WD, we did not observe this difference [14, 15].

A central limitation of any questionnaire‐based study without a contemporaneously interviewed control group is that several assessed symptoms are also common in the general population, so their mere presence cannot be attributed to WD. This is most evident for palpitations, reported by 70% of respondents: palpitations are among the most frequent symptoms in primary care, accounting for roughly 16% of consultations [16, 17]. To place our findings in context, we compared the frequency of the clinically more relevant manifestations against published population‐based estimates. The proportion reporting *frequent* palpitations was low (7%), syncope over 9 years occurred in 7% (vs. a 35% lifetime cumulative incidence in adults aged 35–60 years [18] and 10.5% over 17 years in the Framingham cohort [19]), dizziness in 26% (vs. an annual prevalence of 15%–20% in population‐based studies [20]) and leg oedema in 15% (vs. 19%–20% in adults aged ≥ 51 years [21]). Thus, while non‐specific symptom reporting was—as expected—common, the frequency of clinically relevant cardiac manifestations was comparable to or lower than, that of the general population. Rather than weakening our conclusions, this comparison supports the interpretation that the long‐term cardiac course of treated WD is predominantly benign.

Although not always directly linked to cardiac pathology, respiratory symptoms such as dyspnoea were frequently reported, supporting the notion that such symptoms may emerge or intensify in WD over time. These findings are consistent with previous reports describing diastolic dysfunction and reduced right ventricular function in WD cohorts [4, 5, 22]. Such complaints may reflect subclinical cardiac changes—myocardial fibrosis or diastolic dysfunction—previously identified by cardiac MRI and echocardiography. Although subtle, these abnormalities can contribute to perceived exercise intolerance and fatigue. Indeed, a longitudinal cohort study by Grandis et al. demonstrated a 55% higher risk of heart failure in WD patients compared with controls [6].

An important question raised by the longitudinal design is whether the minor baseline cardiac and autonomic abnormalities predict symptoms years later. In our data, they did not. Across 68 correlations between pre‐specified baseline parameters and patient‐reported outcomes (Table S2), only three reached nominal significance, all in a counter‐intuitive direction and most plausibly attributable to chance and the small number of patients with midwall late gadolinium enhancement (*n* = 5). The absence of a consistent predictive signal suggests that, at the individual‐patient level, subtle baseline imaging or autonomic findings have limited value for forecasting subjective cardiac symptoms over a decade. This does not negate their pathophysiological relevance but argues against their use as stand‐alone prognostic markers and underscores the need for larger cohorts.

Palpitations were nonetheless frequent, aligning with our earlier findings and those of Hlubocká et al. and Batool Hamdani et al., who described electrocardiographic changes such as increased T‐wave dispersion and a higher prevalence of premature ventricular contractions in WD patients [23, 24]. A plausible substrate is diffuse myocardial fibrosis secondary to copper deposition, consistent with autopsy data from Factor et al. documenting copper‐laden fibrotic myocardium and with the cardiac‐MRI study by Salatzki et al. showing increased fibrotic burden in living WD patients [3, 25].

Of concern, only 22% (6/27) of patients remained in regular cardiology care and half of those evaluations revealed unspecified abnormalities. This low follow‐up rate—and the detection of unspecified abnormalities in those who were assessed—suggests that cardiological evaluation should be readily available and considered for WD patients who develop cardiac symptoms or who had abnormal findings on prior imaging [23]. Our data do not, however, establish that fixed‐interval screening of asymptomatic patients improves outcomes, and we therefore refrain from recommending universal periodic cardiac evaluation on this basis. Taken together, while cardiac symptoms and limitations are not uncommon in WD, they generally remain manageable and rarely lead to severe cardiovascular events when patients are adequately treated and monitored.

## 5 Limitations

This study has several caveats. The cohort is small—typical for an orphan disease—yet our 9‐year vital‐status ascertainment of 51% (31/61) approaches the benchmark of 50% often reported in decade‐long follow‐ups [26, 27]. Although the follow‐up subgroup was comparable to the lost‐to‐follow‐up group across nearly all measured baseline variables, survivor and non‐response bias cannot be fully excluded. Most importantly, outcomes were collected exclusively by telephone or emailed questionnaire without a matched, identically interviewed healthy control group; because symptoms such as palpitations, dizziness and dyspnoea are common in the general population, their attribution to WD is inherently limited and we have therefore benchmarked our findings against published population‐based estimates. Re‐interviewing the original imaging‐matched controls was not feasible, as that cohort had been recruited solely for cross‐sectional comparison without consent for longitudinal contact. The questionnaire‐based design also introduces recall bias and precludes objective confirmation of symptoms. Finally, no repeat imaging, ECG, medication‐adherence data or copper‐metabolism indices were obtained, limiting our ability to track subclinical disease progression. Future work should incorporate scheduled cardiac MRI, Holter monitoring, biochemical profiling and—ideally—a prospectively interviewed control group.

## 6 Conclusion

This 9‐year follow‐up study suggests that cardiac symptoms in patients with WD are common but generally mild to moderate in severity. While most participants reported stable or only modestly deteriorating health, a subset experienced symptoms such as shortness of breath, palpitations and dizziness that in some cases impacted daily functioning; however, the frequency of clinically relevant manifestations was comparable to general‐population estimates and baseline imaging and autonomic findings did not predict later symptoms. Severe cardiac events or cardiovascular hospitalisations were not observed, indicating that overt cardiac complications remain relatively rare in well‐managed WD patients. Nonetheless, the persistence of symptoms in some patients and the detection of unspecified findings in those undergoing cardiology assessment support a low threshold for symptom‐guided cardiological evaluation within the interdisciplinary care of WD. Whether systematic screening of asymptomatic patients is beneficial remains unproven and should be addressed by dedicated prospective studies. Given the study's limitations, larger and more comprehensive prospective studies—ideally with a contemporaneous control group—are necessary to better define the trajectory of cardiac involvement in WD and to identify patients at risk of progressive cardiovascular complications.

## Author Contributions

**S. Quick:** conceptualization, methodology, formal analysis, writing – original draft. **M. Cybularz‐Bednarek:** methodology, investigation, data curation. **L. Wiegand:** methodology, investigation, data curation. **F. M. Heidrich:** methodology, investigation, formal analysis, writing – review and editing. **K. Ibrahim:** validation, writing – review and editing. **M. Christoph:** validation, writing – review and editing. **A. Linke:** validation, writing – review and editing. **J. Hamann:** formal analysis, visualization, writing – review and editing. **K. Zhang:** formal analysis, visualization, writing – review and editing.

## Funding

The authors have nothing to report.

## Ethics Statement

Approved by the local ethics committee of the Technische Universität Dresden (BO‐EK‐212052024). All participants provided informed consent.

## Consent

The authors have nothing to report.

## Conflicts of Interest

The authors declare no conflicts of interest.

## Supporting information


**Table S1:** Baseline cardiac magnetic resonance, echocardiographic strain, laboratory and heart rate variability characteristics of the original cohort, the follow‐up subgroup and the patients lost to follow‐up.

**Table S2:** Associations between baseline (2016) cardiac MRI, strain, autonomic and laboratory parameters and patient‐reported outcomes at 9‐year follow‐up.

## Data Availability

The data that support the findings of this study are available from the corresponding author upon reasonable request.
