# Supplementary material for: Cardiac Manifestation in Wilson Disease: Results of a 9‐Year Prospective Cohort
Source: JIMD Rep. 2026 Jun 25;67(4):e70106. doi: 10.1002/jmd2.70106 (PMC13301258; doi:10.1002/jmd2.70106)
Supplement: Supplementary file 1 — Table S1: Baseline cardiac magnetic resonance, echocardiographic strain, laboratory and heart rate variability characteristics of the original cohort, the follow‐up subgroup and the patients lost to follow‐up. [file JMD2-67-e70106-s002.docx]

**Table S1 Baseline cardiac magnetic resonance, laboratory and heart rate variability characteristics of the original cohort, the follow-up subgroup and the patients lost to follow-up**

| **Variable** | **Original cohort (*n* = 61)** | **Follow-up subgroup (*n* = 27)** | **Lost to follow-up (*n* = 34)** | ***p*-value** |
| --- | --- | --- | --- | --- |
| *Left ventricular parameters* |  |  |  |  |
| LV ejection fraction, % | 66.1 (5.0) | 64.5 (4.9) | 67.2 (4.9) | 0.059 |
| LV stroke volume, ml | 81.4 (24.9) | 82.4 (21.7) | 81.4 (26.5) | 0.609 |
| LV end-diastolic volume, ml | 123.7 (38.8) | 128.0 (32.9) | 121.9 (41.8) | 0.271 |
| LV end-systolic volume, ml | 42.1 (15.8) | 45.4 (13.9) | 40.3 (16.5) | 0.140 |
| LV end-diastolic diameter, mm | 49.0 (5.1) | 49.1 (4.9) | 48.5 (5.5) | 0.671 |
| LV mass, g | 114.0 (31.0) | 115.7 (28.1) | 114.0 (33.7) | 0.834 |
| *Late gadolinium enhancement* |  |  |  |  |
| LGE at RV insertion point | 58 (95 %) | — | — | — |
| Midwall LGE | 11 (18 %) | 5 (26 %) | 6 (21 %) | 0.737 |
| *Right ventricular parameters* |  |  |  |  |
| RV ejection fraction, % | 45.7 (3.0) | 46.2 (3.3) | 45.4 (3.1) | 0.379 |
| RV fractional area change, % | 51.7 (4.2) | 51.3 (4.8) | 51.9 (3.8) | 0.617 |
| TAPSE, mm | 24.1 (3.8) | 22.9 (3.6) | 24.8 (3.8) | 0.033 |
| RV stroke volume, ml | 56.9 (15.8) | 57.3 (11.4) | 56.9 (17.4) | 0.506 |
| RV end-diastolic volume, ml | 125.3 (38.0) | 125.5 (27.1) | 125.7 (41.5) | 0.522 |
| RV end-systolic volume, ml | 68.5 (23.0) | 68.0 (17.0) | 69.0 (25.0) | 0.651 |
| RV end-diastolic diameter, mm | 41.2 (6.7) | 40.9 (7.5) | 41.8 (5.7) | 0.645 |
| *Laboratory parameters* |  |  |  |  |
| NT-proBNP, pmol/l | 8.6 (6.8) | 6.3 (4.7) | 10.0 (7.2) | 0.086 |
| Troponin T, ng/l | 4.0 (2.0) | 4.5 (1.2) | 7.7 (8.3) | 0.127 |
| Total serum copper, µmol/l | 6.4 (4.8) | 5.9 (4.4) | 6.7 (5.2) | 0.593 |
| Non-caeruloplasmin-bound serum copper, µmol/l | 2.9 (6.1) | 2.2 (1.6) | 3.5 (8.1) | 0.876 |
| ALT, µmol/(s·l) | 0.7 (0.5) | 0.7 (0.5) | 0.7 (0.5) | 0.955 |
| AST, µmol/(s·l) | 0.6 (0.3) | 0.6 (0.2) | 0.6 (0.4) | 0.767 |
| *Strain* |  |  |  |  |
| RV EndoGLS, % | −23.6 (4.9) | −23.8 (4.8) | −23.4 (5.1) | 0.772 |
| LV MyoGLS (4CV), % | −18.6 (3.5) | −19.1 (3.6) | −18.2 (3.5) | 0.353 |
| LV EndoGLS (4CV), % | −23.3 (4.4) | −23.4 (4.8) | −23.2 (4.0) | 0.849 |
| *Heart rate variability* |  |  |  |  |
| Triangular Index | 30.2 (10.1) | 30.9 (9.2) | 29.7 (10.9) | 0.662 |
| SDNN-Index, ms | 43.7 (28.2) | 40.3 (21.0) | 46.4 (33.0) | 1.000 |

Data are presented as mean (SD) or *n* (%) unless otherwise stated. *p*-values compare the follow-up subgroup with the lost-to-follow-up group; Fisher's exact test for categorical variables, Student's *t*-test for normally distributed continuous variables, Mann-Whitney *U* test for non-normally distributed variables. LV, left ventricular; RV, right ventricular; LGE, late gadolinium enhancement; TAPSE, tricuspid annular plane systolic excursion; NT-proBNP, N-terminal pro-B-type natriuretic peptide; ALT, alanine aminotransferase; AST, aspartate aminotransferase; SDNN, standard deviation of normal-to-normal R-R intervals.
