# Supplementary material for: Cardiac Manifestation in Wilson Disease: Results of a 9‐Year Prospective Cohort
Source: JIMD Rep. 2026 Jun 25;67(4):e70106. doi: 10.1002/jmd2.70106 (PMC13301258; doi:10.1002/jmd2.70106)
Supplement: Supplementary file 2 — Table S2: Associations between baseline (2016) cardiac MRI, strain, autonomic and laboratory parameters and patient‐reported outcomes at 9‐year follow‐up. [file JMD2-67-e70106-s001.docx]

**Table S2 Associations between baseline (2016) cardiac MRI, autonomic and laboratory parameters and patient-reported outcomes at 9-year follow-up**

| **Baseline parameter (2016)** | **General health change** | **Cardiac health change** | **Activity limitation** | **Palpitations frequency** |
| --- | --- | --- | --- | --- |
| *Cardiac MRI parameters* |  |  |  |  |
| LV ejection fraction, % | +0.10 (0.63) | −0.03 (0.90) | −0.10 (0.64) | −0.08 (0.70) |
| LV mass, g | −0.32 (0.13) | −0.15 (0.50) | +0.10 (0.66) | +0.17 (0.45) |
| LV end-diastolic volume, ml | −0.05 (0.83) | −0.16 (0.47) | +0.19 (0.39) | +0.15 (0.50) |
| RV ejection fraction, % | +0.06 (0.80) | −0.06 (0.79) | −0.26 (0.23) | −0.04 (0.87) |
| TAPSE, mm | −0.04 (0.87) | −0.20 (0.35) | −0.17 (0.45) | −0.05 (0.83) |
| RV end-diastolic volume, ml | −0.19 (0.38) | −0.03 (0.91) | +0.18 (0.42) | +0.03 (0.87) |
| LGE burden (% myocardium) | +0.24 (0.33) | +0.42 (0.07) | −0.05 (0.82) | −0.10 (0.69) |
| Midwall LGE (yes/no) | −0.20 (0.42) | +0.07 (0.78) | **−0.54 (0.02)** | **−0.46 (0.05)** |
| *Strain* |  |  |  |  |
| RV EndoGLS, % | **−0.42 (0.04)** | −0.40 (0.06) | +0.03 (0.88) | +0.34 (0.11) |
| LV MyoGLS (4CV), % | +0.03 (0.89) | +0.12 (0.58) | +0.24 (0.27) | +0.19 (0.38) |
| LV EndoGLS (4CV), % | +0.13 (0.55) | +0.12 (0.59) | +0.34 (0.12) | +0.10 (0.66) |
| *Autonomic function (24-h Holter)* |  |  |  |  |
| SDNN-Index, ms | −0.01 (0.98) | −0.22 (0.33) | +0.21 (0.36) | +0.12 (0.59) |
| Triangular Index | +0.01 (0.97) | −0.33 (0.14) | −0.20 (0.38) | +0.08 (0.72) |
| *Cardiac biomarkers* |  |  |  |  |
| NT-proBNP, pmol/l | +0.30 (0.17) | +0.06 (0.78) | +0.04 (0.87) | +0.08 (0.72) |
| Troponin T, ng/l | −0.24 (0.27) | −0.04 (0.86) | +0.14 (0.53) | +0.15 (0.51) |
| *Wilson disease severity* |  |  |  |  |
| UWDRS total score | +0.17 (0.42) | +0.13 (0.53) | +0.27 (0.21) | +0.06 (0.77) |
| UWDRS neurologic score | +0.06 (0.78) | −0.05 (0.83) | +0.12 (0.59) | +0.16 (0.45) |

Spearman rank correlation coefficients *(ρ)* with corresponding *p*-values are shown for the 27 patients with completed 9-year follow-up. Bold values indicate *p* < 0.05. LV, left ventricular; RV, right ventricular; LGE, late gadolinium enhancement; TAPSE, tricuspid annular plane systolic excursion; SDNN, standard deviation of normal-to-normal R-R intervals; NT-proBNP, N-terminal pro-B-type natriuretic peptide; UWDRS, Unified Wilson Disease Rating Scale.
